# Generation of Functional Oligodendrocyte Progenitor Cells Through Serial Replating of iPSC-Derived NPC Spheres

**DOI:** 10.3390/cells15121067

**Published:** 2026-06-11

**Authors:** Junmyeong Park, Seungye Kang, Soojin Kim, Donghyun Kim, Borami Shin, Ji Young Mun, Yurim Park, Johnny Kim, Steven A. Goldman, Kee-Pyo Kim

**Affiliations:** 1 Department of Medical Life Sciences, College of Medicine, The Catholic University of Korea, 222 Banpo-daero, Seocho-gu, Seoul 06591, Republic of Korea; parkjunmai@catholic.ac.kr (J.P.); yeah403@catholic.ac.kr (S.K.); susantina@catholic.ac.kr (S.K.); dbdxmrdhfl@catholic.ac.kr (D.K.); 2 Department of Medical Sciences (Graduate School), College of Medicine, The Catholic University of Korea, 222 Banpo-daero, Seocho-gu, Seoul 06591, Republic of Korea; 3 Department of General Pediatrics, University of Children’s Hospital Muenster, Albert-Schweitzer-Campus 1, Gebäude A1, 48149 Münster, Germany; 4 Neural Circuits Research Group, Korea Brain Research Institute (KBRI), 61 Cheomdan-ro, Dong-gu, Daegu 41062, Republic of Korea; 5 The Center for Cardiovascular Regeneration and Immunology at TRON—Translational Oncology at the University Medical Center of the Johannes Gutenberg-University Mainz gGmbH, Freiligrathstraße 12, 55131 Mainz, Germany; 6 Vascular Biology and Therapeutics Program, Department of Comparative Medicine, Yale University School of Medicine, 333 Cedar Street, New Haven, CT 06510, USA; 7 Center for Translational Neuromedicine, University of Rochester Medical Center, 601 Elmwood Ave, Rochester, NY 14642, USA; 8 Faculty of Health and Medical Sciences, University of Copenhagen, Blegdamsvej 3B, 2200 Copenhagen, Denmark

**Keywords:** iPSCs, neural progenitor cells, oligodendrocyte progenitor cells, oligodendrocytes

## Abstract

**Highlights:**

**What are the main findings?**

- Stage-specific modulation of developmental signaling in iNPCs enabled the efficient production of OPCs within a reduced culture duration.
- Serial replating of iNPC-derived spheres allowed the sustained generation of CD140a^+^/O4^+^ OPC populations.

**What are the implications of the main findings?**

- The shortened OPC differentiation duration enables scalable research and disease modeling.
- Sustained OPC generation via serial replating serves as a robust and scalable platform, ensuring a continuous cell source for cell-based remyelination therapies.

**Abstract:**

Oligodendrocytes (OLs) are essential for myelin formation in the central nervous system, and their loss or dysfunction is a hallmark of various demyelinating and neurodegenerative disorders. Although oligodendrocyte precursor cells (OPCs) represent a promising cell source for remyelination therapies, existing protocols for generating OPCs from human-induced pluripotent stem cells (iPSCs) are often limited by prolonged culture duration, low efficiency, and cellular heterogeneity. Here, we report an efficient and reproducible platform for generating OPCs from iPSC-derived neural progenitor cells (iNPCs) through stage-specific modulation of developmental signaling pathways. Directed differentiation of iNPCs recapitulated key developmental transitions, progressing through OLIG2^+^/NKX2.2^+^ progenitors to CD140a^+^/O4^+^ OPCs within a significantly shortened timeframe compared to conventional approaches. Notably, iNPC-derived spheres functioned as a progenitor-like niche, enabling sustained OPC production through serial replating. Purified OPCs could differentiate into MBP^+^ oligodendrocytes and demonstrated myelination capacity both in vitro, via nanofiber ensheathment and in vivo following transplantation into shiverer (shi/shi) mice, where they formed myelin sheaths around host axons. Despite these advances, OPC differentiation and maturation efficiencies remained suboptimal, highlighting the need for further optimization. Collectively, our findings establish a scalable and time-efficient strategy for iPSC-derived OPC generation and underscore their potential for disease modeling and cell-based remyelination therapies.

## 1. Introduction

Oligodendrocytes (OLs) are specialized glial cells in the central nervous system responsible for the formation and maintenance of myelin sheaths, which insulate axons and facilitate efficient signal transmission [1,2]. During early brain development, OLs are generated at relatively late stages from oligodendrocyte precursor cells (OPCs), a distinct progenitor population [3]. Both OLs and OPCs are critical for proper neuronal function and the overall structural and functional integrity of the nervous system [2]. Beyond serving as a reservoir for mature OLs, OPCs have emerged as active contributors to neuronal communication and plasticity, underscoring their importance in brain development as well as in neurodevelopmental disorders [4,5]. In addition, accumulating evidence implicates OLs and OPCs in the pathogenesis of various neurological diseases, including neurodegenerative disorders [6,7,8].

Demyelinating diseases, such as multiple sclerosis, leukodystrophies, and Pelizaeus–Merzbacher Disease, are characterized by the progressive loss of these myelin sheaths, leading to severe neurological deficits and axonal degeneration [1,9]. While current pharmacological interventions, primarily immunomodulators, are effective in retarding disease progression by suppressing inflammation, they fail to restore the loss of oligodendrocytes and myelin [10,11]. Given these limitations, cell-based therapies aimed at replacing oligodendrocytes and promoting remyelination have emerged as a promising alternative approach [12,13]. In this context, OPCs are considered a more suitable candidate than fully mature OLs due to their migratory capacity, ability to engraft within the host central nervous system, and potential to ensheath host axons, thereby restoring functional conductivity [14].

Substantial efforts have been made to generate OPCs from induced pluripotent stem cells (iPSCs) as a renewable and clinically relevant cell source [15,16,17,18,19]. Over the past decade, several protocols have been developed to direct the differentiation of human iPSCs into the oligodendrocyte lineage [15,16,17,18,19]. However, a major limitation of these approaches is the prolonged culture duration, often requiring 75 to 150 days to generate O4^+^ oligodendrocytes. In addition, many protocols exhibit low differentiation efficiency and cellular heterogeneity, often leading to contamination by non-neural lineages. Although transcription factor-driven strategies have significantly accelerated lineage specification, concerns regarding random genomic integration and associated safety risks limit their translational potential [20,21,22,23]. Therefore, there remains a critical need for a safe, defined, and time-efficient strategy to generate high-purity OPCs suitable for therapeutic applications.

In this study, we established an efficient and reproducible platform for generating OPCs from human iPSC-derived NPCs (iNPCs). Our differentiation strategy faithfully recapitulates key developmental transitions, progressing from NPCs to OLIG2^+^/NKX2.2^+^ lineage-committed cells and ultimately to CD140a^+^/O4^+^ OPCs, while markedly reducing the overall culture duration compared to previously reported protocols. Notably, repeated rounds of OPC production were achieved through serial replating of iNPC-derived spheres, allowing sustained generation of CD140a^+^/O4^+^ OPCs. The derived OPCs readily differentiated into myelin basic protein (MBP)-positive cells and exhibited the ability to ensheathe nanofibers, indicative of functional myelination capacity. Importantly, upon transplantation into the forebrain of neonatal immunocompromised shiverer (shi/shi) mice, these cells exhibited robust engraftment and formed myelin sheaths around host axons, demonstrating in vivo myelination capacity. Collectively, these findings support the feasibility of generating functional OPCs through serial replating of iPSC-derived NPC spheres and highlight their potential utility for cell-based remyelination therapies.

## 2. Materials and Methods

### 2.1. Induced Pluripotent Stem Cell Culture

CRL-2097 neonatal skin fibroblasts were purchased from ATCC (Manassas, VA, USA) and reprogrammed into hiPSCs, as described in a previous study [24]. Human iPSCs were maintained on Matrigel-coated plates with MEF-conditioned medium (MEF-CM), prepared as described previously [24]. The medium was replaced daily, and cells were passaged every 4–5 days using TrypLE™ Express (Gibco, Grand Island, NY, USA). All cultures were maintained at 37 °C with 5% CO_2_ in a humidified BB15 incubator (Thermo Fisher Scientific, Waltham, MA, USA).

### 2.2. Derivation of Neural Progenitor Cells from iPSCs

iPSC-derived NPCs (iNPCs) were derived from iPSCs by adapting previously described protocols [25,26]. For this, iPSCs were dissociated into single cells using TrypLE™ Express and resuspended in hESC medium without bFGF, containing 10 µM SB431542 (SB, Stemcell, Vancouver, BC, Canada), 250 nm LDN193189 (LDN, Stemcell, Vancouver, BC, Canada), 0.5 µM SAG (agonist of the SHH pathway, TargetMol, Boston, MA, USA), and 3 µM CHIR99021 (CHIR, AdooQ, Irvine, CA, USA) and 10 µM Y27632 (AdooQ, Irvine, CA, USA). The hESC medium without bFGF was DMEM/F12 (Corning, New York, NY, USA) supplemented with 20% KnockOut Serum Replacement (Gibco, Grand Island, NY, USA), 1 mm 2-mercaptoethanol (Gibco, Grand Island, NY, USA), 1X non-essential amino acids (NEAA, WELGENE, Gyeongsan, Republic of Korea), 1X L-glutamine (Sigma-Aldrich, Burlington, MA, USA), and 1X penicillin/streptomycin (P/S, WELGENE, Gyeongsan, Republic of Korea). Approximately 30,000 cells per well were seeded into a 96-well V-bottom plate (Sarstedt, Numbrecht, Germany), centrifuged at 1000 rpm for 5 min at room temperature and incubated at 37 °C with 5% CO_2_ for 48 h to form embryoid bodies (EBs). On day 2, the EBs were transferred to ultra-low attachment plates (Corning, New York, NY, USA) and cultured in NPC medium containing 10 µM SB, 250 nm LDN, 0.5 µM SAG and 3 µM CHIR. The NPC medium was a 1:1 mixture of DMEM/F12 and Neurobasal (Gibco, Grand Island, NY, USA) supplemented with 0.5X N2 (Gibco, Grand Island, NY, USA), 0.5X B-27 minus vitamin A (Gibco, Grand Island, NY, USA), 1X P/S, and 1X L-glutamine. On day 4, SB and DOR were withdrawn and 150 µM ascorbic acid (AA, Sigma-Aldrich, Burlington, MA, USA) was added to the NPC medium. On day 6, the EBs were mechanically dissociated into small aggregates. The dissociated clumps were then plated on Matrigel-coated plates. After 48 h of attachment, cells were passaged using Accutase (Sigma-Aldrich, Burlington, MA, USA) at a 1:5 ratio. Thereafter iNPCs were cultured with the NPC medium containing 0.5 µM SAG, 3 µM CHIR and 150 µM AA and passaged every 3–4 days at a 1:10 ratio.

### 2.3. Cumulative Growth Assay

iNPCs were cultured under the conditions described above. Three independent clones were analyzed for 36 days. Cells were passaged every 4 days and plated at 1 × 10^6^ cells per passage. At each passage, total cell numbers were determined using a hemocytometer and trypan blue. Cumulative cell growth was calculated based on the total number of cells obtained at each time point.

### 2.4. Generation of Oligodendrocytes from iPSC-Derived NPCs

Protocols for iPSC to oligodendrocyte (OL) differentiation have been previously reported [15,16,18]. We modified and applied this to the iNPC-to-OL protocol. iNPCs were plated on Matrigel-coated dishes that reached >90% confluence (Day 0) and were cultured with OPC medium supplemented with 100 nM retinoic acid (RA, Sigma-Aldrich, Burlington, MA, USA) and 1 µM SAG (agonist of the SHH pathway). The OPC medium was DMEM/F12, supplemented with 1X N-2, 1X B-27 minus vitamin A, 50 µM 2-mercaptoethanol, 1X NEAA, 1X L-glutamine, 1X P/S, and 25 µg/mL insulin (Sigma-Aldrich, Burlington, MA, USA). To ensure sustained signaling for lineage specification, the medium supplemented with 100 nM RA and 1 µM SAG was replenished daily. On day 4, cells were mechanically detached using a cell scraper to generate small clumps and transferred to ultra-low attachment plates (Corning, New York, NY, USA) and maintained in OPC medium supplemented with 100 nM RA and 1µM SAG until day 11, with medium changes every other day. From day 12 to 21, RA and SAG were withdrawn from the medium and the clumps were cultured in OPC medium supplemented with 10 ng/mL platelet-derived growth factor AA (PDGF-AA, Peprotech, Cranbury, NJ, USA), 10 ng/mL insulin-like growth factor 1 (IGF 1, Peprotech, Cranbury, NJ, USA), 5 ng/mL hepatocyte growth factor (HGF, Peprotech, Cranbury, NJ, USA), 10 ng/mL neurotrophin 3 (NT-3, Peprotech, Cranbury, NJ, USA), 100 ng/mL biotin (Sigma-Aldrich, Burlington, MA, USA), and 10 µM cyclic Adenosine MonoPhosphate (cAMP, Sigma-Aldrich, Burlington, MA, USA). The medium was changed every other day. On day 22, clumps of 200–500 µm in diameter were selected and plated onto culture plates pre-coated with 50 µg/mL poly-L-ornithine (Sigma-Aldrich, Burlington, MA, USA) and 10 µg/mL laminin (Sigma-Aldrich, Burlington, MA, USA) and the medium was changed every other day. On day 48, PDGF-AA, IGF 1, HGF, and NT3 were withdrawn and 60 ng/mL triiodothyronine (T3, Sigma-Aldrich, Burlington, MA, USA) and 20 µg/mL ascorbic acid (AA) were added.

### 2.5. In Vitro Myelination Assay

Co-culturing with synthetic nanofibers (Nanofiber solutions, Columbus, OH, USA) was performed as previously described [27,28,29]. Briefly, CD140a^+^O4^+^ cells were seeded at a density of 5 × 10^4^ cells/well onto poly-L-ornithine/laminin-coated nanofibers in a 4-well plate and cultured with 60 ng/mL T3 for 5 days.

### 2.6. In Vivo Transplantation

All surgical interventions and presurgical and postsurgical animal care were provided in accordance with the Laboratory Animals Welfare Act, the Guide for the Care and Use of Laboratory Animals and the Guidelines and Policies for Rodent Survival Surgery provided by the IACUC (Institutional Animal Care and Use Committee) in the School of Medicine, The Catholic University of Korea (Approval number: CUMC-2025-0006-02).

Transplantation was performed as previously described [15,30,31]. Homozygous shiverer mice were crossed with homozygous rag2 null immunodeficient mice to generate a line of shi/shi × rag^2−/−^ myelin-deficient and immunodeficient mice. Neonatal pups at postnatal days 1–3 (P1–3) were cryoanesthetized on ice for approximately 2–3 min. A total of 3 × 10^5^ cells were suspended in HBSS and distributed across five distinct injection sites using a Hamilton syringe. Specifically, 5 × 10^4^ cells in 0.5 μL of HBSS were microinjected bilaterally into each of the anterior and posterior regions of the corpus callosum. Additionally, 1 × 10^5^ cells/μL were targeted to the cerebellar peduncle. At 10 weeks post-transplantation, mice were anesthetized with isoflurane. Perfusion was performed using DPBS followed by 4% PFA (Biosesang, Yongin, Republic of Korea). The brains were subsequently removed and immersed in 4% PFA for post-fixation.

### 2.7. Immunohistochemistry of Brain Section

Brains were cryosectioned at 20–25 μm. Sections were permeabilized and blocked simultaneously in DPBS containing 1% BSA, 5% normal donkey serum, and 0.1% Triton X-100 for 1 h 30 min at room temperature. Sections were then incubated overnight at 4 °C with primary antibodies diluted in DPBS containing 1% BSA and 0.1% Triton X-100. After three 10 min washes in DPBS, sections were incubated with Alexa Fluor-conjugated secondary antibodies (1:1000; Thermo Fisher Scientific, Waltham, MA, USA) for 2 h at room temperature. Nuclei were counterstained with DAPI (1:1000) for 10 min, and sections were mounted with Fluoromount-G (SouthernBiotech, Birmingham, AL, USA). Images were acquired using a Zeiss LSM 910 confocal microscope. Images were analyzed with Zen lite.

The following primary antibodies were used: anti-human nuclear antigen (1:100, ab191181, Abcam, Cambridge, UK), anti-MBP (1:100, MAB386, Millipore, Burlington, MA, USA), anti-neurofilament 160/200 (1:200, N2912, Sigma-Aldrich, Burlington, MA, USA), anti-β III-tubulin (TUJ1, 1:200, 802001, BioLegend, San Diego, CA, USA), anti-MAG (1:100, 45268, Cell Signaling Technology, Danvers, MA, USA), anti-MOG (1:200, 851702, BioLegend, San Diego, CA, USA), anti-SOX10 (1:200, AF2864, R&D Systems, Minneapolis, MN, USA), and anti-PLP1 (1:200, sc-23570, Santa Cruz Biotechnology, Dallas, TX, USA).

### 2.8. FACS

Differentiated cells were dissociated into a single-cell suspension using Accutase (Sigma-Aldrich, Burlington, MA, USA) for 10 min at 37 °C. All dissociated cells from the cultures were collected without selecting specific clusters or regions. The cells were resuspended in 3% FBS/PBS (100 µL per 1 × 10^6^ cells) and incubated for 10 min at room temperature to block non-specific binding. After centrifugation at 1500 rpm for 3 min, the cell pellet was resuspended in 3% FBS/PBS and incubated with the following antibodies for 30 min on ice: APC-conjugated mouse anti-human/mouse/rat O4 (1:100, 130-119-155, Miltenyl Biotec, Bergisch Gladbach, Germany) and PE-conjugated mouse anti-human CD140a (1:20, 556002; BD Biosciences, Franklin Lakes, NJ, USA). The cells were then washed three times with 3% FBS/PBS and passed through a 5 mL polystyrene round-bottom tube equipped with a cell strainer cap (SPL Life Sciences, Pocheon, Republic of Korea) to remove aggregates. DAPI was added to the final suspension to distinguish live from dead cells. Flow cytometry was performed using FACSCanto II (BD Biosciences, Franklin Lakes, NJ, USA) and cell sorting was performed using FACSAria III (BD Biosciences, Franklin Lakes, NJ, USA). Single cells were separated from debris and aggregates by FSC/SSC gating strategy. Dead cells were excluded by gating on DAPI-negative cells. Single-stain controls were used for compensation adjustment, and unstained cells served as negative controls to determine background fluorescence. Data were analyzed using FlowJo softwarev10.10.0 (BD Biosciences, Franklin Lakes, NJ, USA).

### 2.9. Immunofluorescence

Cells were fixed with 10% formalin (Fujifilm Corporation, Tokyo, Japan) for 20 min at room temperature, permeabilized with 0.1% Triton X-100 in DPBS for 30 min, and blocked in 5% BSA/PBS for 1 h. The cells were then incubated with appropriate primary antibodies diluted in 1% BSA overnight at 4 °C: anti-OCT4 (1:1000, 5677, Cell Signaling Technology, Danvers, MA, USA), anti-SSEA4 (1:100, 330402, BioLegend, San Diego, CA, USA), anti-TRA-1-60 (1:100, 560071, BD Biosciences, Franklin Lakes, NJ, USA), anti-NANOG (1:1000, 5232S, Cell Signaling Technology, Danvers, MA, USA), anti-PAX6 (1:500, 901301, BioLegend, San Diego, CA, USA), anti-SOX1 (1:200, AF3369, R&D Systems, Minneapolis, MN, USA), anti-NESTIN (1:500, 60091, STEMCELL Technologies, Vancouver, BC, Canada), anti-SOX2 (1:1000, 5024S, Cell Signaling Technology, Danvers, MA, USA), anti-GFAP (1:200, Z033429-2, Agilent Technologies, Santa Clara, CA, USA), anti-OLIG2 (1:500, AB9610, Millipore, Burlington, MA, USA), anti-MAP2 (1:500, MAB3418, Millipore, Burlington, MA, USA), anti-S100B (1:100, 90393s, Cell Signaling Technology, Danvers, MA, USA), anti-TUBB3 (1:500, 801202, BioLegend, San Diego, CA, USA), anti-NKX2.2 (1:50, 74.5A5, DSHB, Iowa City, IA, USA), anti-SOX10 (1:100, AF2864, R&D Systems, Minneapolis, MN, USA), anti-PDGFRα (1:800, 5241S, Cell Signaling Technology, Danvers, MA, USA), anti-NG2 (1:500, 554275, BD Biosciences, Franklin Lakes, NJ, USA), anti-O4 (1:100, MAB345, Millipore, Burlington, MA, USA), anti-MBP (1:100, MAB386, Millipore, Burlington, MA, USA), and anti-PLP1 (1:500, sc-23570, SantaCruz, Dallas, TX, USA). Following three washes with DPBS, the cells were incubated with appropriate fluorescently labeled Alexa-Fluor secondary antibodies (1:1000, Thermo Fisher Scientific, Waltham, MA, USA) for 1 h at room temperature. After three additional washes, nuclei were counterstained with DAPI for 10 min and washed once with DPBS. Images were acquired using an inverted fluorescence microscope (IX71 or IX73; Olympus, Tokyo, Japan) equipped with either a CCD (DP30BW; Olympus, Tokyo, Japan) or a CMOS camera (DP23M; Olympus, Tokyo, Japan). Confocal images were acquired with an LSM910 w/Airyscan II confocal microscope. Images were analyzed with Zen lite, cellSens standard and Fiji software (ImageJ version 1.53t).

### 2.10. Quantitative PCR

Total RNA was isolated using the NucleoSpin RNA Mini Kit (MACHEREY-NAGEL, Dueren, Germany) and reverse-transcribed with the iScript™ cDNA Synthesis Kit (Bio-Rad, Hercules, CA, USA). Quantitative real-time PCR (qPCR) was conducted using iTaq™ Universal SYBR^®^ Green Supermix (Bio-Rad, Hercules, CA, USA) on a QuantStudio™ 5 Real-Time PCR System (Applied Biosystems, Foster City, CA, USA). Relative gene expression levels were normalized to *RPL37A* and calculated using the 2^−ΔΔCt^ method with QuantStudio™ Design & Analysis Software v1.5.2. All reactions were performed in triplicate; primer sequences are listed in Table S1.

### 2.11. Tissue Preparation for Transmission Electron Microscopy (TEM) Analysis

The brain was fixed in 2% paraformaldehyde and 2.5% glutaraldehyde in 0.15 M sodium cacodylate buffer (pH 7.4) at 4 °C and sectioned into 120 μm thick slices using a vibratome (VT1000S, Leica, Nussloch, Germany). Tissue slices were post-fixed in a solution containing 2% osmium tetroxide (OsO4; 19150, Electron Microscopy Sciences, Hatfield, PA, USA) and 1.5% potassium ferrocyanide (60279, Sigma-Aldrich, Burlington, MA, USA) in 0.15 M sodium cacodylate buffer for 1 h on ice, followed by washing with distilled water. The samples were incubated in 1% thiocarbohydrazide solution (Tokyo Chemical Industry, Tokyo, Japan) for 20 min and 2% OsO4 for 30 min in distilled water at room temperature. After washing, samples were stained overnight with 1% uranyl acetate (22400, Electron Microscopy Sciences, Hatfield, PA, USA) at 4 °C. The following day, samples were dehydrated through a graded ethanol series (20%, 50%, 70%, 90%, and 100%) and then placed in 100% acetone for 10 min. Dehydrated samples were sequentially infiltrated with acetone/epoxy resin mixtures at ratios of 3:1, 1:1, and 1:3, followed by incubation in 100% resin. Samples were embedded in epoxy resin using the Embed-812 embedding kit (14120Electron Microscopy Sciences, Hatfield, PA, USA) according to the manufacturer’s instructions and polymerized at 60 °C for 3 days. For transmission electron microscopy (TEM) imaging, 60 nm thick ultrathin sections were prepared using an ultramicrotome (EM UC7, Leica Microsystems, Nussloch, Germany) equipped with a diamond knife (40-US, Diatome, Nidau, Switzerland) and mounted on copper slot grids with a specimen support film. Ultrathin sections were observed using a Tecnai G2 transmission electron microscope (FEI) operated at 120 kV and equipped with a US1000X-P camera.

### 2.12. Statistical Analysis

All data were presented as mean ± SD. Statistical analyses were performed using GraphPad Prism version 8 (GraphPad Software, San Diego, CA, USA). Comparisons between two groups were performed using a two-tailed Student’s *t*-test, and comparisons among three groups were performed using one-way ANOVA followed by Tukey’s multiple comparisons test. A *p*-value < 0.05 was considered statistically significant. The number of samples (*n*) for each experiment is indicated in the corresponding figure legends.

## 3. Results

### 3.1. Generation of iPSC-Derived NPCs via Stage-Specific Signaling Modulation

**Figure 1:**
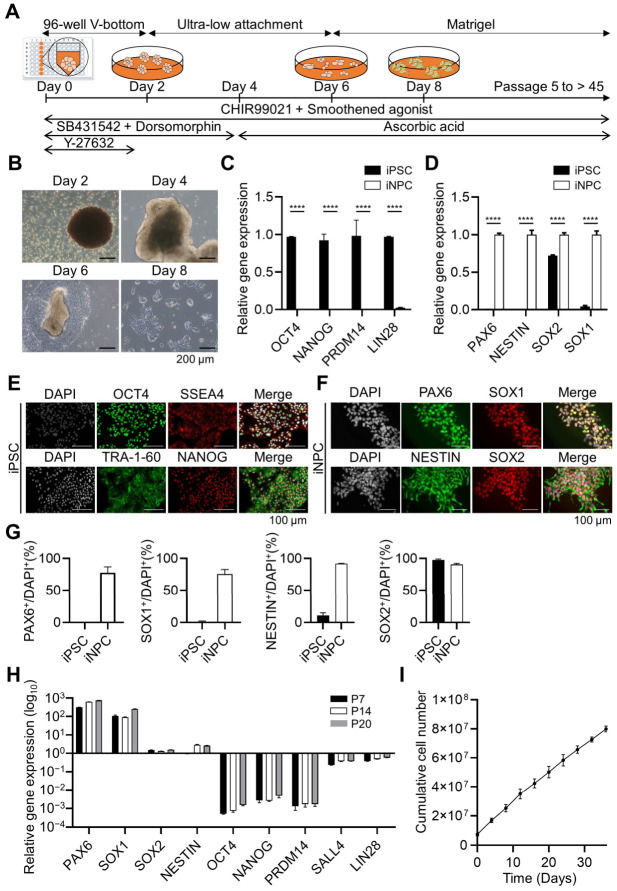
Generation and characterization of iPSC-derived neural progenitor cells. (**A**) Schematic overview of the iNPC generation from iPSC. (**B**) Morphological images from embryoid bodies (EBs) to neuroectodermal lineage cells. Scale bars: 200 μm. (**C**) Relative expression of pluripotency-associated genes (*OCT4*, *NANOG*, *PRDM14*, and *LIN28*) and (**D**) NPC genes (*PAX6*, *SOX1*, *SOX2*, and *NESTIN*) (*n* = 3). Expression values of these genes were normalized by the expression value of RPL37A. **** *p* < 0.0001 (two-tailed *t*-test). (**E**) Immunofluorescence analysis of pluripotency (OCT4, SSEA4, TRA1-60, and NANOG) in iPSCs and (**F**) NPC (PAX6, SOX1, SOX2, and NESTIN) markers in iNPCs. Scale bars: 100 μm. (**G**) Quantification of cells positive for NPC markers (PAX6, SOX1, SOX2, and NESTIN) in iPSCs and iNPCs (*n* = 3). DAPI was used as a nuclear counterstain. (**H**) Relative expression of NPC genes (PAX6, SOX1, SOX2, and NESTIN) and pluripotent genes (OCT4, NANOG, PRDM14, SALL4, and LIN28) across p7, p14 and p20. Statistical analysis was performed on log_10_ data. Expression values of these genes were normalized by the expression value of RPL37A and expressed relative to iPSCs (value = 1). (**I**) Proliferative kinetics of iNPCs over a 35-day expansion period (*n* = 3). All data are presented as mean ± SD.

As NPCs can serve as a cellular source for generating myelinogenic oligodendrocytes [25], we first generated NPCs from iPSCs using stage-specific small molecules and growth factors that recapitulate developmental signaling pathways involved in neurogenesis (Figure 1A). Briefly, iPSCs were dissociated, replated into 96-well V-bottom plates (30,000 cells per well), and centrifuged to form embryoid bodies (EBs) (day 0) (Figure 1A). The EBs were then cultured in NPC medium supplemented with CHIR99021 (CHIR; a GSK-3 inhibitor), SAG (agonist of the SHH pathway), SB431542 (SB; a TGF-β receptor inhibitor), dorsomorphin (DOR; a BMP inhibitor), and Y-27632 (Y; a ROCK inhibitor), promoting specification toward the neuroectodermal lineage. After 2 days, individual EBs were manually transferred to low-attachment plates and maintained in NPC medium containing CHIR, SAG, SB, and DOR (Figure 1A,B). On day 4, SB and DOR were withdrawn, and ascorbic acid (AA) was added instead to promote further neuroectodermal differentiation (Figure 1A,B). On day 6, EBs were mechanically dissociated into smaller aggregates and plated onto Matrigel-coated plates to allow adherent monolayer growth (Figure 1A,B). From day 6 onward, cells were cultured in NPC medium supplemented with CHIR, SAG, and AA, and passaged enzymatically every 72–96 h. After five consecutive passages, cells were cryopreserved in liquid nitrogen and subsequently characterized as iPSC-derived NPCs (hereafter referred to as iNPCs).

### 3.2. Defining Cellular and Molecular Identity of iNPCs

To define the cellular identity of derived iNPCs, we performed immunofluorescence and qPCR analyses. qPCR analyses showed that pluripotency-associated genes, including *OCT4*, *NANOG*, *PRDM14*, and *LIN28*, were markedly downregulated in iNPCs (Figure 1C). In contrast, NPC genes, such as *PAX6*, *NESTIN*, and *SOX1*, were significantly upregulated (Figure 1D). In the case of SOX2, its expression was detected in both iPSCs and iNPCs. Consistent with the qPCR data, immunofluorescence analysis revealed that OCT4, SSEA4, TRA1-60, and NANOG were robustly expressed in iPSCs but not in iNPCs (Figure 1E and Supplementary Figure S1A). Conversely, NPC markers, such as PAX6, SOX1, and NESTIN, were highly expressed in iNPCs (Figure 1F–G and Supplementary Figure S1B). SOX2 expression persisted in both cell types. Importantly, iNPCs maintained stable proliferation and cellular identity during in vitro prolonged culture, as confirmed by qPCR and cumulative cell-counting analyses. Specifically, no significant changes were observed in the expression of NPC markers (*PAX6*, *SOX1*, *SOX2*, and *NESTIN*), while pluripotency markers (*OCT4*, *NANOG*, *PRDM14*, *SALL4*, and *LIN28*) remained suppressed across serial passages (p7 to p20) (Figure 1H). Furthermore, cumulative cell-counting analysis demonstrated that iNPCs were robustly expandable for over 30 days without loss of their proliferative capacity (Figure 1I). Taken together, these data suggest that the iNPCs generated in this study are expandable and sustainable and exhibit molecular characteristics similar to those of their resident counterparts in human brain tissue [32].

### 3.3. Evaluation of In Vitro Differentiation Potential of iNPCs

**Figure 2:**
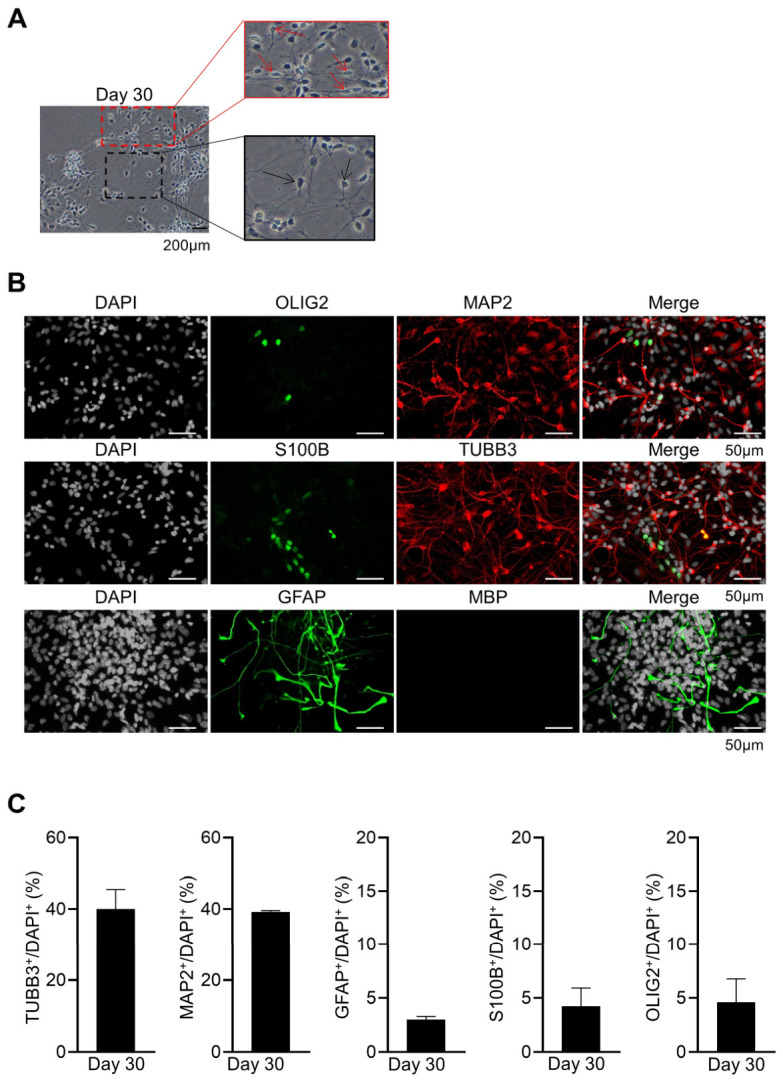
Multipotency of iNPCs and their differentiation into neurons, astrocytes, and oligodendrocytes. (**A**) Morphology of spontaneously differentiated iNPCs on day 30, showing cells with neuronal-like (dendritic and axonal) morphology (red arrows) and glial-like morphology (black arrows). Scale bars: 200 μm. (**B**) Immunofluorescence analysis of MAP2^+^, β-Tubulin III (TUBB3)^+^, GFAP^+^, S100B^+^, MBP^+^ and OLIG2^+^ in differentiated iNPCs. Scale bars: 50 μm. (**C**) Quantification of MAP2^+^, β-Tubulin III (TUBB3)^+^, GFAP^+^, S100B^+^, MBP^+^ and OLIG2^+^ cells (*n* = 6). DAPI was used as a nuclear counterstain. All data are presented as mean ± SD.

NPCs are multipotent stem cells that are capable of differentiating into neurons, astrocytes, and oligodendrocytes [25,33]. To assess the in vitro differentiation potential of iNPCs, we cultured the cells in NPC medium lacking mitogens and other factors that support iNPC self-renewal for 30 days. We observed morphological changes in differentiated cells, some of which showed dendritic and axonal morphology, while others displayed glial-like morphology (Figure 2A). To further characterize the cellular identity of these differentiated cells, we stained them with antibodies against MAP2, β-Tubulin III (TUBB3), GFAP, S100B, MBP, and OLIG2. β-Tubulin III (TUBB3) and MAP2 are markers for immature and mature neurons [34]. GFAP and S100B can label astrocytes [35]. OLIG2 is a marker for pan-oligodendroglial cells [16,36]. MBP can only detect mature myelinating oligodendrocytes [37]. We observed that TUBB3- and MAP2-positive cells were relatively abundant (~40%), indicating the neuronal differentiation propensity of iNPCs (Figure 2B,C). GFAP- and S100B-positive astrocytes accounted for 2.9 ± 0.31% and 4.2 ± 1.7% of the population, respectively, while 4.5 ± 2.1% of cells were OLIG2-positive (Figure 2B,C). Unlike other makers, we were unable to detect MBP-positive cells, suggesting that the current differentiation conditions are insufficient to support full oligodendrocyte maturation and that additional signaling cues are required to recapitulate the later stages of oligodendrocyte lineage progression (Figure 2B). Taken together, these results demonstrate that iNPCs generated in this study exhibit multipotency such that they could differentiate into neurons, astrocytes, and glial progenitor cells, but with limited capacity to generate fully mature oligodendrocytes under the current conditions.

### 3.4. Generation of OPCs from iNPCs Using Developmental Signaling Cues

**Figure 3:**
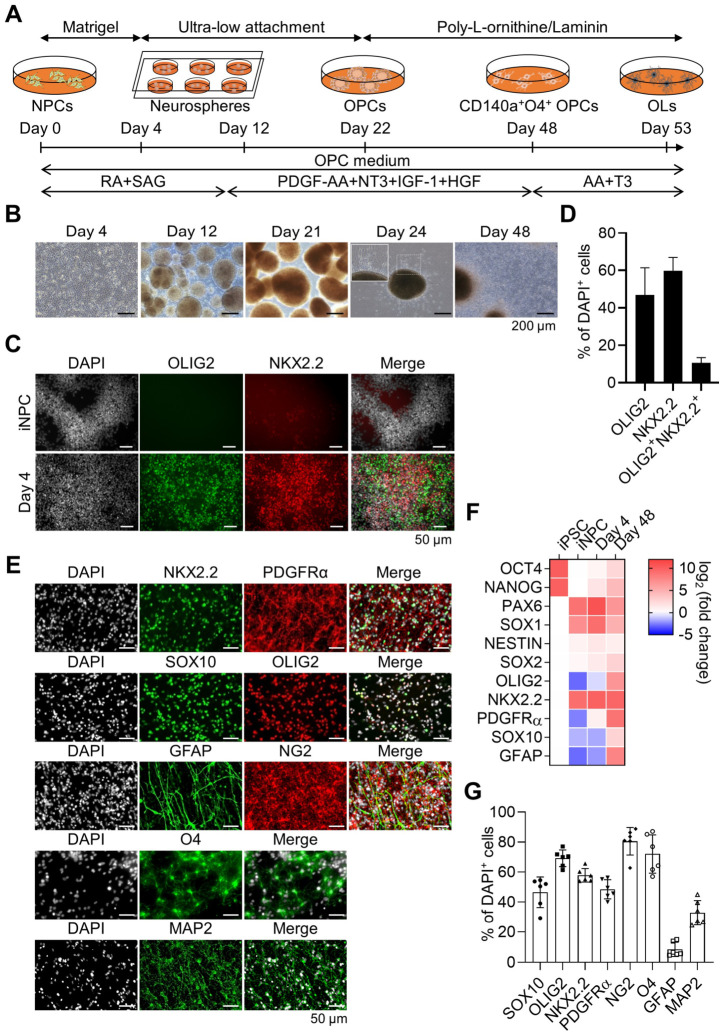
Differentiation of OPCs from iNPCs in the presence of mitogens over a 48-day period. (**A**) Schematic of the differentiation protocol into mature oligodendrocytes. (**B**) Morphological changes during differentiation of iNPCs. Scale bars: 200 μm. (**C**) Immunofluorescence analysis of OLIG2^+^ and NKX2.2^+^ cells on day 4. Scale bars: 50 μm. (**D**) Quantification of OLIG2^+^, NKX2.2^+^ and OLIG2^+^/NKX2.2^+^ cells (*n* = 5). DAPI was used as a nuclear counterstain. (**E**) Immunofluorescence analysis of OPC (NKX2.2, PDGFRα, SOX10, OLIG2, NG2, and O4), astrocyte (GFAP) and neuron (MAP2) markers on day 48. Scale bars: 50 μm. (**F**) Heatmap showing normalized expression of pluripotency (*OCT4*, *NANOG*), NPC (*PAX6*, *SOX1*, *SOX2*, and *NESTIN*), OPC (*OLIG2*, *NKX2.2*, *PDGFRα*, *SOX10*), and astrocyte (*GFAP*) genes during the differentiation process (*n* = 3). Expression values of these genes were normalized by expression value of *RPL37A* and represented as log_2_ fold changes. (**G**) Quantification of cells positive for OPC (NKX2.2, PDGFRα, SOX10, OLIG2, NG2, and O4), astrocyte (GFAP) and neuron (MAP2) markers (*n* = 6, indicated by dots). DAPI was used as a nuclear counterstain. All data are presented as mean ± SD.

To promote differentiation of iNPCs toward oligodendrocyte progenitor cells (OPCs) and functionally mature oligodendrocytes, iNPCs were plated onto Matrigel-coated plates and cultured in OPC medium supplemented with retinoic acid (RA; a metabolite of vitamin A) and SAG (day 0) (Figure 3A). On day 4, cells were mechanically dissociated into small clumps using a cell scraper and transferred to an ultra-low attachment plate (Figure 3A). These clumps were then cultured in suspension in the presence of RA and SAG. During continued culture until day 12, these clumps increased in size and became more spherical as progenitor cells proliferated, usually reaching a diameter of 200–300 μm within 8 days (Figure 3A,B). On day 12, RA and SAG were withdrawn from the medium, and platelet-derived growth factor AA (PDGF-AA), hepatocyte growth factor (HGF), insulin-like growth factor 1 (IGF1), and neurotrophin 3 (NT3), which are known to be mitogens for OPCs [15,18], were added to the medium (Figure 2A). On day 22, we transferred these spheres to a poly-L-ornithine/laminin-coated plate to facilitate outgrowth, expansion, and maturation of OPCs (Figure 3A,B). The outgrown cells were then enzymatically dissociated and subjected to fluorescence-activated cell sorting (FACS) to isolate CD140a^+^ and O4^+^ cells, representing a putative OPC population. Sorted cells were subsequently replated onto poly-L-ornithine/laminin-coated plates and cultured in the presence of triiodothyronine (T3) to promote terminal differentiation (Figure 3A).

NPCs undergo a series of morphological and molecular changes during differentiation into terminally mature oligodendrocytes [18,37]. To assess the progression of cell differentiation, we monitored morphological changes and marker expression. By day 4, iNPCs self-organized into radial structures, and a substantial fraction of cells expressed OLIG2 and NKX2.2 (Figure 3B,C). Quantitative analysis revealed that more than 40% of cells were OLIG2-positive, while over 50% expressed NKX2.2 (Figure 3D). OLIG2- and NKX2.2-coexpressing cells exhibit a phenotype reminiscent of specialized neuroepithelial cells in the developing ventral spinal cord, which play a critical role in oligodendrocyte differentiation and patterning [3]. Combined RA and SAG treatment has been shown to induce OLIG2^+^/NKX2.2^+^ neuroepithelial cells from NPCs [18,36]. In line with these notions, RA and SAG treatment induced a transition of iNPCs toward an OLIG2^+^/NKX2.2^+^ neuroepithelial-like state, albeit with lower efficiency (~10 ± 2.8%) (Figure 3D). By day 24, cells with small, dark cell bodies and branched processes began to emerge at the periphery of the spheres (Figure 3B). These cells migrated outward, proliferated actively, and progressively increased in number until day 48 (Figure 3B). Once the outgrowth reached confluence, cultures were terminated and the cells were characterized by immunofluorescence and qPCR analyses. We found that NKX2.2, PDGFRα, SOX10, OLIG2, NG2, and O4, which are oligodendroglial markers [15], were expressed in outgrown cells at day 48 (Figure 3E). Consistent with these findings, *OLIG2*, *SOX10*, and *PDGFRα*, which were not expressed in iNPCs, were highly upregulated in day 48 outgrowth OPCs (Figure 3F). These data suggest that the outgrown cells from the spheres are indeed bona fide OPCs. However, not all cells expressed OPC markers, and a subset of cells expressed SOX10 (46.5 ± 10.2%), OLIG2 (69.1 ± 5.1%), NKX2.2 (57.8 ± 4.4%), PDGFRα (48.5 ± 6.4%), NG2 (80.5 ± 9.2%), and O4 (72 ± 12.7%) (Figure 3G). Moreover, only 39.6 ± 7.9% of cells were double-positive for SOX10 and OLIG2. A fraction of outgrown cells also expressed GFAP (8.5 ± 4.6%) and MAP2 (32.7 ± 8.1%), indicating the presence of astrocytic and neuronal populations (Figure 3E–G). Collectively, these data suggest that the protocol developed in this study yields heterogeneous cell populations in which OPCs, GPCs, neurons, and astrocytes simultaneously emerge during in vitro differentiation [38]. Therefore, an additional purification step is a prerequisite to isolate a homogeneous OPC population that is capable of differentiation into mature myelinating oligodendrocytes.

### 3.5. Serial Replating of Spheres Sustains OPC Production with Limited Self-Renewal Capacity

**Figure 4:**
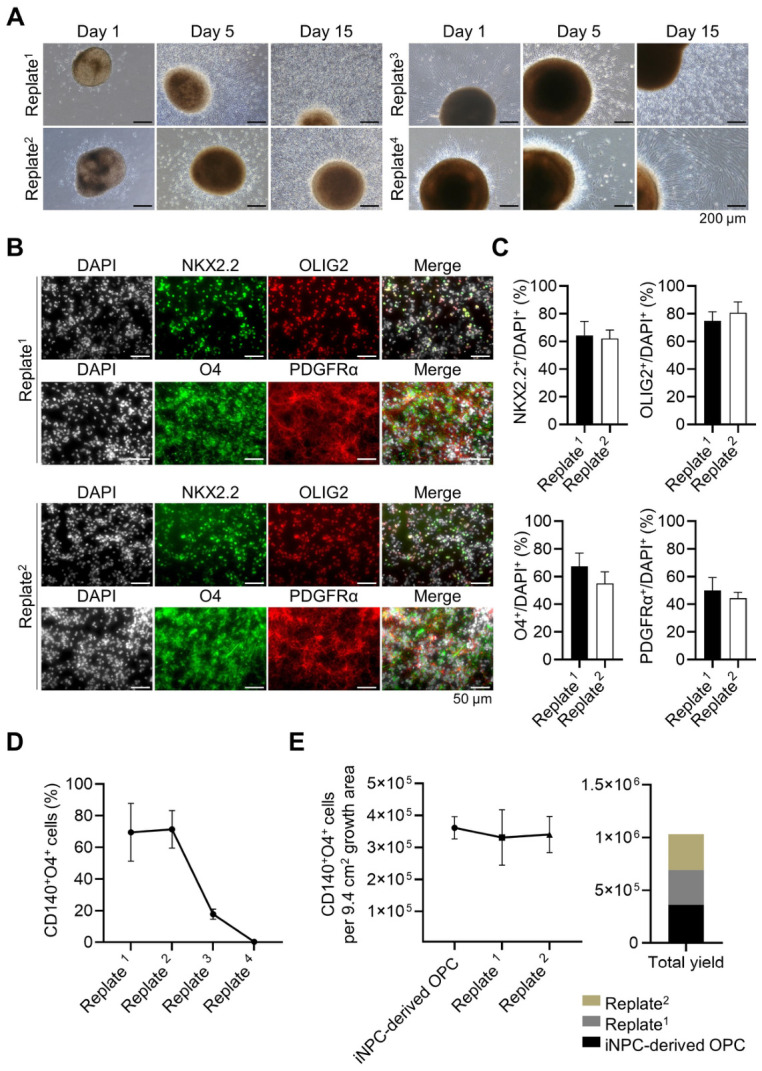
Continuous generation of iNPC-derived OPCs through repeated sphere replating. (**A**) Morphological changes in outgrowth cells from spheres during serial replating on poly-L-ornithine/laminin-coated plates on days 1, 5 and 15. Scale bars: 200 μm. (**B**) Immunofluorescence analysis of NKX2.2^+^, OLIG2^+^, O4^+^, and PDGFRα^+^ cells on day 15 of the first and second replating. Scale bars: 50 μm. (**C**) Quantification of NKX2.2^+^, OLIG2^+^, O4^+^, PDGFRα^+^ cells (*n* = 4). DAPI was used as a nuclear counterstain. (**D**) Flow cytometry analysis showed CD140a^+^/O4^+^ cell proportions over four successive replating steps (*n* = 3). (**E**) Quantification of CD140a^+^/O4^+^ cells in a 9.4 cm^2^ culture area and cumulative OPC yield over two successive replatings (*n* = 3). Replate^1^ to Replate^4^ indicate the first to fourth rounds of serial replating. All data are presented as mean ± SD.

The core of the spheres appeared to function as a progenitor niche, likely harboring stem or precursor cells that maintained a constant volume while continuously generating proliferative outgrowth cells (Figure 3B). Notably, even at the terminal stage of culture (usually days 42–48), sphere size remained largely unchanged (Figure 3B). Based on this observation, we hypothesized that mechanical transfer and replating of spheres could enable sustained production of proliferating OPCs. To test this hypothesis, spheres were mechanically lifted up using a pulled glass pipette and replated onto a new poly-L-ornithine/laminin-coated plate. By the following day, the replated spheres had stably attached, and OPCs began to migrate outward from the attached spheres (Figure 4A). These OPCs proliferated actively and reached confluence within 15 days (Figure 4A). Importantly, the majority of these cells expressed OPC markers, including NKX2.2 (64.36 ± 10%), OLIG2 (74.9 ± 6.4%), O4 (67.4 ± 9.4%), and PDGFRα (49.9 ± 9.3%), indicating that transfer and replating of spheres did not alter the fate of the outgrown cells (Figure 4B,C). We then performed a second round of sphere replating once the initial outgrowth reached confluence. Interestingly, outgrown OPC cells expressing NKX2.2 (62 ± 6.1%), OLIG2 (80.5 ± 7.9%), O4 (55 ± 8.3%), and PDGFRα (44.3 ± 4.2%) were again successfully produced following this second round of replating (Figure 4B,C). However, subsequent replating beyond this point (the third round onward) failed to yield OPC populations, as the proportion of CD140a^+^/O4^+^ cells was markedly reduced (Figure 4D). Nevertheless, during multiple rounds of replating, we obtained over 1 × 10^6^ purified CD140a^+^/O4^+^ OPCs per 9.4 cm^2^ of the culture surface, yielding approximately 3.5 × 10^5^ cells at each replating step (Figure 4E). These findings suggest that the progenitor niche within the spheres possesses a limited capacity for sustained OPC production, which becomes progressively exhausted upon repeated mechanical passaging.

### 3.6. OPCs Differentiate into Myelinating Oligodendrocytes In Vitro and In Vivo

**Figure 5:**
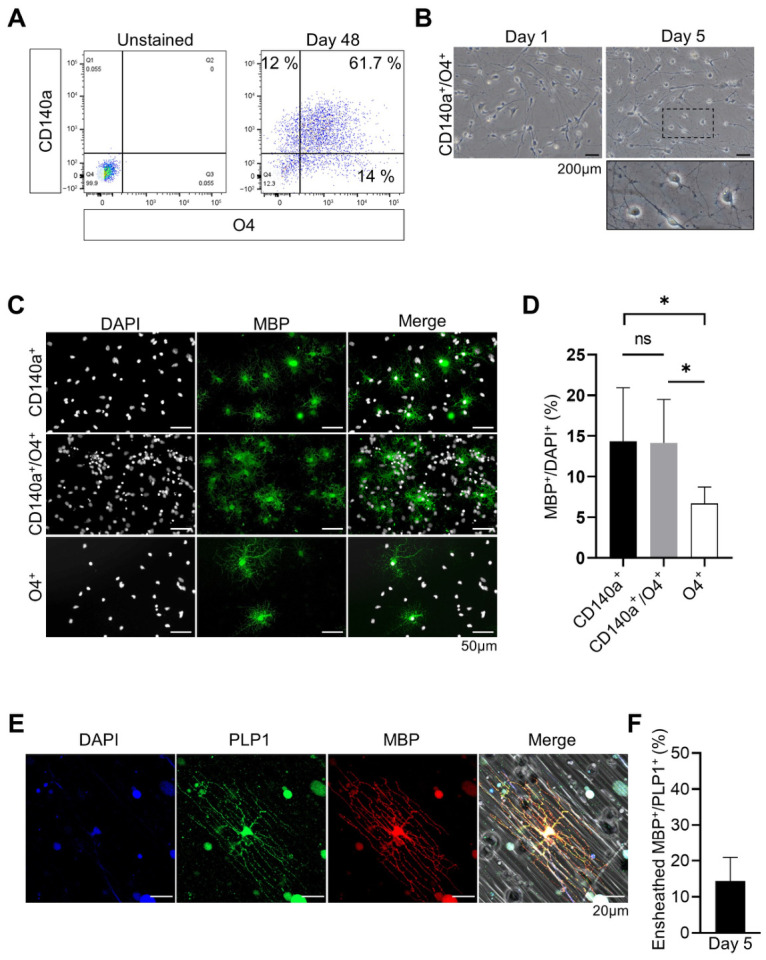
In vitro and in vivo differentiation of iNPC-derived OPCs into mature and myelinating oligodendrocytes. (**A**) Representative FACS plots for CD140a and O4 at day 48. Their expression was analyzed by flow cytometry. (**B**) Morphological change from OPC to mature oligodendrocyte. The dotted box indicates the area enlarged below, showing multiple elongated and highly branched processes. Scale bars: 200 μm. (**C**) Immunofluorescence analysis of MBP^+^ cells in CD140a^+^, CD140a^+^/O4^+^ and O4^+^ cells at day 5 of maturation. DAPI was used as a nuclear counterstain. Scale bar: 50 μm. (**D**) Quantification of MBP^+^ cells in CD140a^+^, CD140a^+^/O4^+^ and O4^+^ cells (*n* = 4). * *p* < 0.05; ns, not significant (one-way ANOVA). (**E**) Immunofluorescence analysis of MBP^+^ and PLP1^+^ cells ensheathing nanofibers merged with bright field and DAPI. Scale bars: 20 μm. (**F**) Quantification of ensheathed MBP^+^/PLP1^+^ cells on nanofibers (*n* = 6). DAPI was used as a nuclear counterstain.

We next tested whether OPCs generated through serial replating of iPSC-derived NPC spheres can be differentiated into mature oligodendrocytes. Outgrown cells were enzymatically dissociated and subjected to fluorescence-activated cell sorting (FACS) to isolate CD140a^+^, O4^+^, or CD140a^+^/O4^+^ populations (Figure 5A). Sorted cells were then cultured in a medium supplemented with ascorbic acid (AA) and triiodothyronine (T3) for 5 days to promote further differentiation. T3 is widely used to induce OPC differentiation into oligodendrocytes [18,39,40]. During this period, cells exhibiting a small, oval-shaped soma with 2–3 thin bipolar processes developed into cells with multiple elongated and highly branched processes, consistent with the characteristic morphology of mature oligodendrocytes (Figure 5B). To further define their cellular identity, we stained them with antibodies against MBP. MBP is a gold standard for identifying actively myelinating oligodendrocytes [41]. We observed MBP-expressing cells, indicating that OPCs can differentiate into mature oligodendrocytes in response to T3 (Figure 5C). Of note, only ~15% of cells were MBP-positive (CD140a^+^: 14.3 ± 6.5%, CD140a^+^/O4^+^: 14.1 ± 5.3%, O4^+^: 6.7 ± 2%), suggesting that the overall differentiation efficiency remains low under the current culture conditions, despite prior enrichment of a specific antigen-positive population (Figure 5D). To assess the myelinating potential of iNPC-derived OPCs, cells were co-cultured with nanofibers for 5 days in the presence of T3. OPCs gave rise to MBP^+^/PLP1^+^ oligodendrocytes with segmented MBP and PLP1 tracts in close apposition to nanofibers, indicative of axonal ensheathment and myelinating capacity (Figure 5E,F).

**Figure 6:**
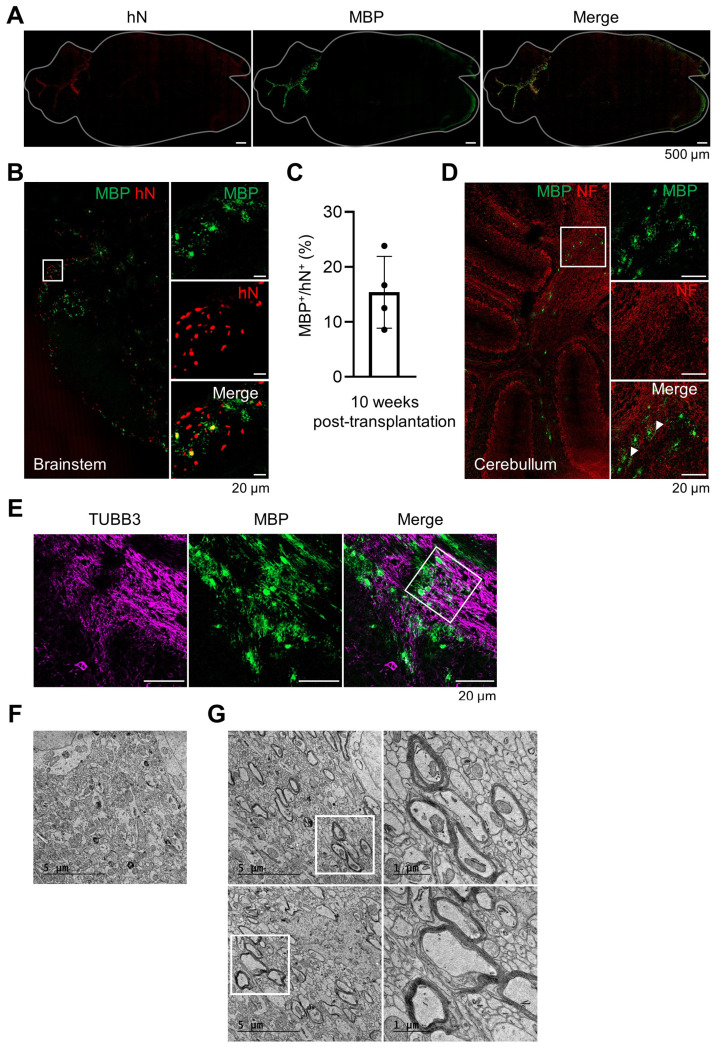
In vivo engraftment and myelination of iNPC-derived OPCs in shiverer mice. (**A**) Representative sagittal tile-scan images of an engrafted brain at 10 weeks post-transplantation, immunostained for hN and MBP. Scale bar: 500 μm. (**B**) Immunohistochemistry analysis of MBP^+^ cells among hN^+^ donor cells. The boxed region is shown at higher magnification. Scale bars: 20 μm. (**C**) Quantification of MBP^+^ cells among hN^+^ donor cells at 10 weeks post-transplantation (*n* = 4 mice). Data are presented as mean ± SD. (**D**) Immunohistochemistry analysis of donor-derived MBP^+^ profiles and host axons (NF). The boxed region is shown at higher magnification; arrowheads indicate MBP^+^ ensheathment. Scale bars: 20 μm. (**E**) Immunohistochemistry analysis of donor-derived MBP^+^ profiles ensheathing host axons (TUBB3), confirming axonal ensheathment with an independent axonal marker. The red boxed region highlights an area of MBP^+^ ensheathment. Scale bars: 20 μm. (**F**,**G**) Representative electron microscopic images of the striatum of an engrafted shiverer mouse at 10 weeks post-transplantation. (**F**) A region without myelination, showing unmyelinated axons. (**G**) A region containing myelinated axons, showing compact, multilayered myelin sheaths. The boxed regions are shown at higher magnification on the right. Scale bars: 5 μm (**F** and **G left**) and 1 μm (**G right**).

To further evaluate myelinating potential of iNPC-derived OPCs in vivo, day 48 outgrown cells were transplanted without further sorting into neonatal (P1–P3) immunodeficient shiverer (shi/shi × rag2^−^/^−^) mice. We used a five-site injection protocol targeting the anterior and posterior corpus callosum bilaterally and the cerebellar peduncle, delivering a total of 3 × 10^5^ cells per mouse [15,30,31]. At 10 weeks post-transplantation, tile-scanning of sagittal sections immunolabeled for human nuclei (hN) and MBP revealed that donor-derived cells had dispersed from the injection sites and engrafted predominantly in the white matter of the brainstem and cerebellum (Figure 6A). Because shiverer mice lack functional MBP, the observed MBP^+^ cells were necessarily donor cells [15,30]. At 10 weeks post-transplantation, 15.4 ± 6.5% of hN^+^ donor cells expressed MBP (*n* = 4 mice) (Figure 6B, C). Donor-derived MBP^+^ profiles formed segmented structures that aligned with and ensheathed host axons, as confirmed by co-labeling with the axonal markers neurofilament (NF) and TUBB3 (Figure 6D). The engrafted cells also expressed the additional oligodendroglial markers PLP1, MOG, MAG, and SOX10, with donor-derived expression confirmed by co-localization with hN, or with donor-specific MBP for MAG (Supplementary Figure S2A). Finally, to evaluate myelin formation at the ultrastructural level, we examined the striatum of engrafted shiverer mice at 10 weeks post-transplantation using electron microscopy (EM). Compared to regions lacking myelination (Figure 6F), compact, multilayered myelin sheaths ensheathing host axons were frequently observed in the engrafted areas (Figure 6G). Together, these results indicate that iNPC sphere-derived OPCs engraft and differentiate into myelinating oligodendrocytes that ensheath host axons in vivo.

## 4. Discussion

In this study, we established a reproducible platform for generating NPCs from human iPSCs through stage-specific modulation of developmental signaling pathways (Figure 1). Although the resulting iPSC-derived NPCs exhibited stable proliferation and maintained canonical NPC marker expression over extended passages (Figure 1H,I), their precise regional identity and functional equivalence to in vivo human NPCs remain to be fully defined. Future studies incorporating region-specific patterning cues, such as rostrocaudal and dorsoventral signaling gradients, as well as transcriptomic analyses, will be important to more precisely characterize and refine iNPC identity and lineage potential.

We directed the differentiation of iNPCs into OPCs by recapitulating key developmental signaling pathways (Figure 3). Although this approach significantly reduced the time required for OPC generation, the resulting cultures remained heterogeneous, necessitating additional purification steps (Figure 3E–G). To address this limitation, future strategies should focus on improving lineage specificity through optimized combinations of small molecules or transient, non-integrating transcriptional modulation approaches that enhance oligodendrocyte lineage commitment without compromising genomic safety. In addition, the development of chemically defined culture systems will further improve the purity and scalability of OPC production, while reducing the overall time required for OPC generation.

A key finding of this study is that NPC-derived spheres function as a progenitor-like niche capable of sustaining OPC production through serial replating (Figure 4). However, the observed decline in OPC yield upon repeated replating indicates a limited self-renewal capacity of the progenitor pool (Figure 4D). This constraint could be addressed by modulating niche-supporting signals, such as growth factor gradients and extracellular matrix composition, to better preserve progenitor competence. Alternatively, engineered 3D microenvironments could be explored to enhance scalability and maintain long-term OPC production capacity in a more controlled and reproducible manner.

Although iNPC-derived OPCs were capable of differentiating into MBP^+^ oligodendrocytes and demonstrated myelination capacity in vitro and in vivo, the overall efficiency of terminal differentiation remained relatively low, even after enrichment of an OPC population (Figure 5C,D). This suggests that current differentiation conditions are insufficient to fully support synchronized and efficient maturation into myelinating oligodendrocytes. In vivo, engrafted cells were concentrated in the white matter of the brainstem and cerebellum (Figure 6A), and underwent oligodendroglial differentiation, as evidenced by expression of MBP, PLP1, MOG, MAG, and SOX10 (Figure 6B,D and Supplementary Figure S2A). Their differentiation into other neural lineages was not examined in detail and remains an important direction for future study. The regional density of engrafted cells was also not quantified, owing to their non-uniform distribution. Moreover, our in vivo analysis was limited to a single time point; longitudinal studies will be needed to clarify how engraftment, distribution, and oligodendroglial differentiation evolve over time. While no abnormal growth was observed following transplantation, long-term safety, stability, and functional integration remain to be systematically evaluated. Collectively, these limitations highlight the need for further optimization of differentiation cues, expansion strategies, and maturation conditions to fully realize the therapeutic potential of iPSC-derived OPCs.

## 5. Conclusions

In summary, we established a reproducible and time-efficient system for generating human OPCs from iPSC-derived NPCs through a serial replating strategy. These OPCs differentiated into MBP^+^ oligodendrocytes that ensheathed nanofibers in vitro and axons in vivo, forming compact myelin in the engrafted brain. This approach provides a scalable platform that, with continued refinement, holds promise not only for disease modeling and drug screening but also as a potential foundation for cell-based therapies targeting demyelinating disorders.

## Data Availability

The original contributions presented in this study are included in the article/Supplementary Material. Further inquiries can be directed to the corresponding authors.

## Supplementary Materials

The following supporting information can be downloaded at https://www.mdpi.com/article/10.3390/cells15121067/s1. Figure S1: Immunofluorescence analysis of pluripotency and NPC marker in iNPCs and iPSCs as negative controls; Figure S2: Immunohistochemistry analysis of oligodendroglial markers in engrafted iNPC-derived OPCs; Table S1: List of primers used for qPCR.
