# Supplementary material for: Generation of Functional Oligodendrocyte Progenitor Cells Through Serial Replating of iPSC-Derived NPC Spheres
Source: Cells. 2026 Jun 11;15(12):1067. doi: 10.3390/cells15121067 (PMC13297427; doi:10.3390/cells15121067)
Supplement: Supplementary file 1 [file cells-15-01067-s001.zip › cells-4278673-Supplementary.pdf]

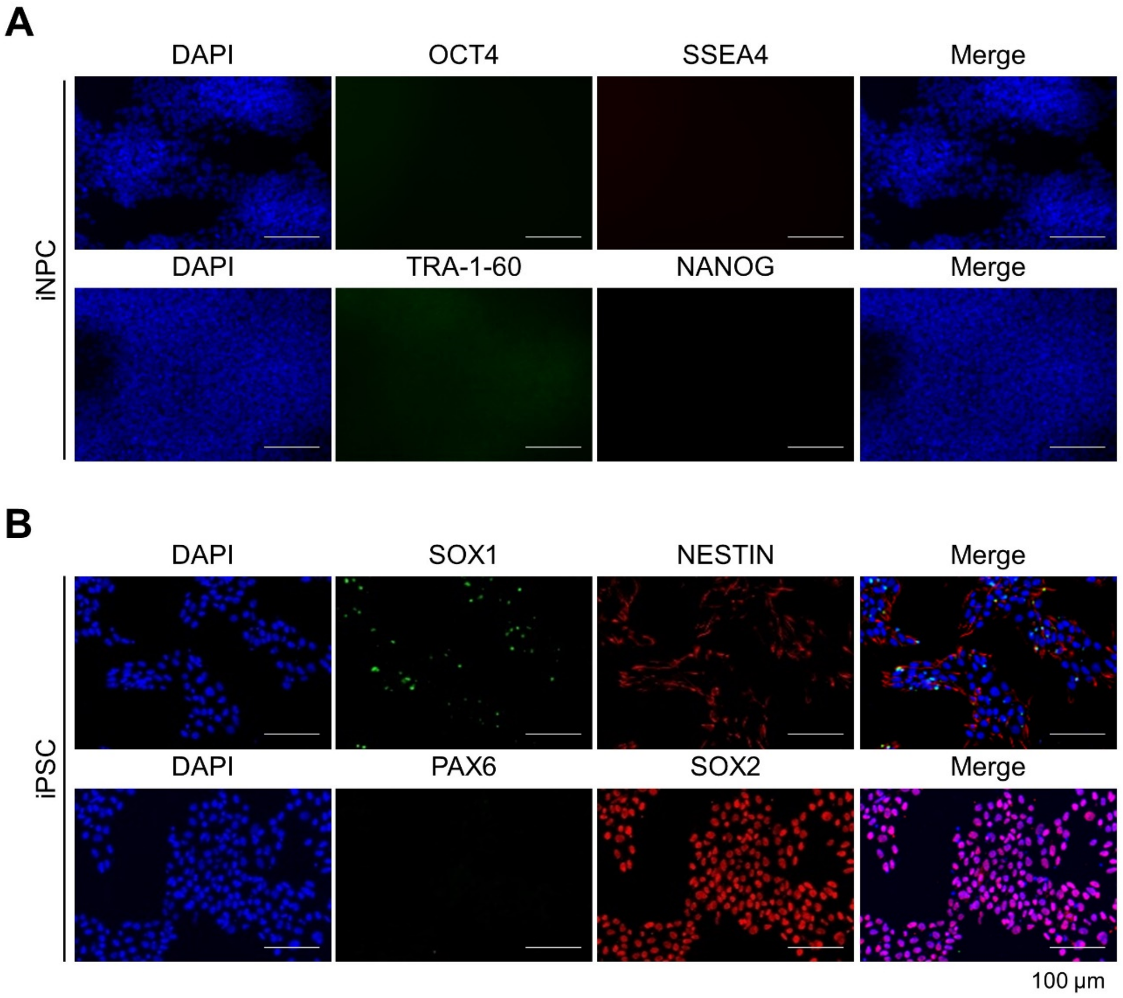

Figure S1. Immunofluorescence analysis of pluripotency and NPC marker in iNPCs and iPSCs as negative controls. (A) Representative images of iNPCs stained with pluripotency markers (OCT4, SSEA4, TRA1-60, and NANOG), showing no dectable expression. Scale bars: 100 μm. (B) Representative images of iPSCs stained with NPC markers (PAX6, SOX1, SOX2, and NESTIN), showing no dectable expression. Scale bars: 100 μm.

A

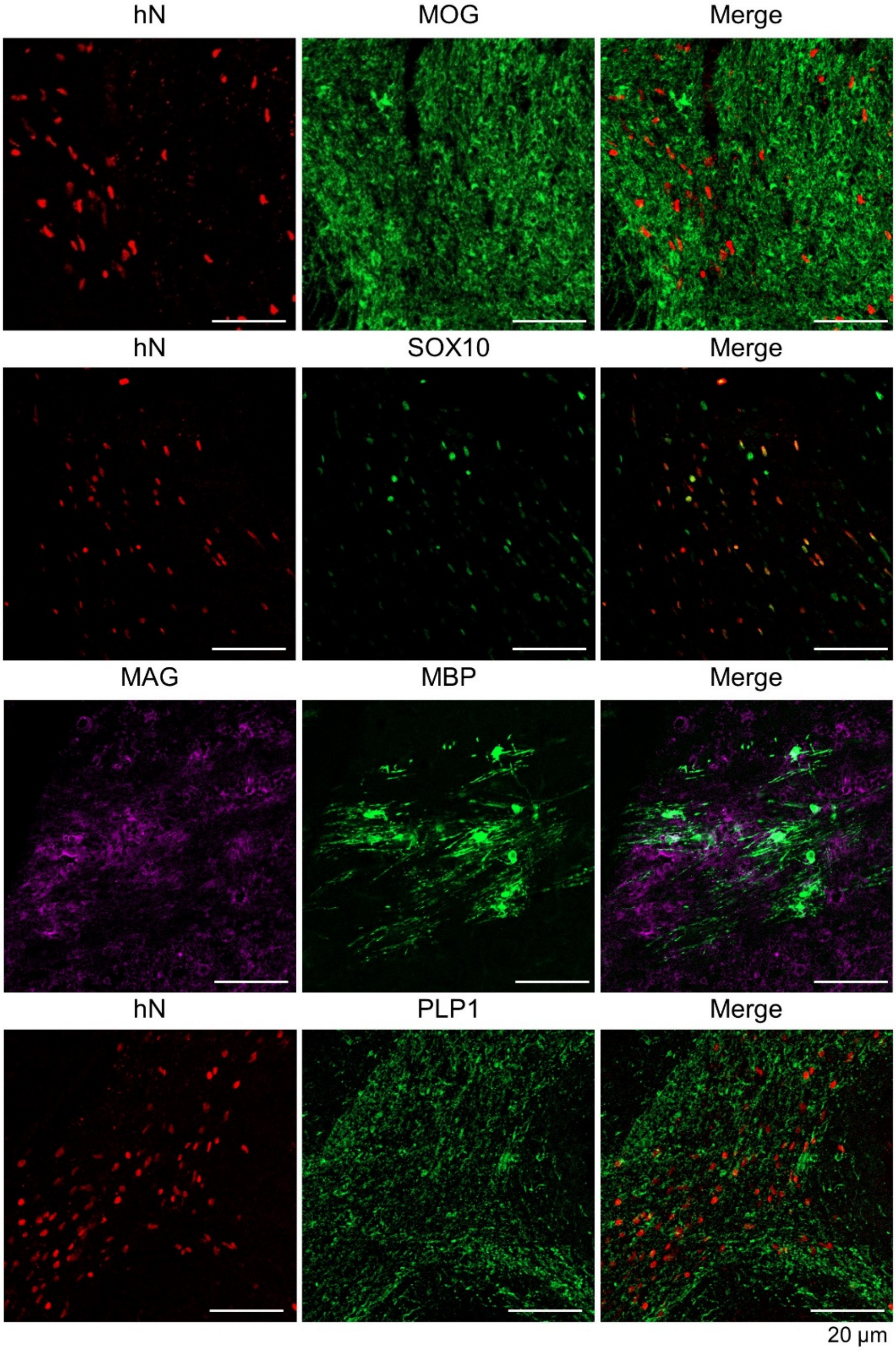

Figure S2. Immunohistochemistry analysis of oligodendroglial markers in engrafted iNPC-deriv

ed OPCs. (A) Representative images of donor-derived (hN+) cells stained with the oligodendro glial markers MOG, SOX10, MAG, and PLP1. MAG was co-labeled with MBP. Scale bars: 20 μm

Table S1: List of primer used for qPCR.

Gene	Primer sequence	
OCT4	F	TGGTCCGAGTGTGGTTCTGTAA
	R	TGTGCATAGTCGCTGCTTGAT
NANOG	F	CCAAAGGCAAACAACCCACTT
	R	TCTTGACCGGGACCTTGTCT
SALL4	F	CACATTTGTGGGACCCTCGACATTG
	R	CGCCCCGTGTGTCATGTAGTGAACC
PRMD14	F	CATGCGAGTCCACTCTGGAGAC
	R	CTGATGTGTGTGCGGAGTATGC
LIN28	F	GGAGGCCAAGAAAGGGAATATGA
	R	AACAATCTTGTGGCCACTTTGACA
PAX6	F	TCGGGCACCACTTCAACAG
	R	TCCGGGAACTTGAACTGGAA
NESTIN	F	CTG CTA CCC TTG AGACACCTG
	R	GGG CTC TGA TCT CTGCATCTAC
SOX2	F	CCA ACG GTG TCA ACC TGC AT
	R	GGA AGG AAT TGG GAA CAC AAA GG
SOX1	F	CAGCAGTGTCGCTCCAATTCA
	R	GCCAAGCACCGAATTCACAG
OLIG2	F	CAC AGA GCA GTG GGG AGT G
	R	GCA CAC AGC GGT ACC TTT TC
NKX2.2	F	GAC AAC TGG TGG CAG ATT TCG CTT
	R	AGC CAC AAA GAA AGG AGT TGG ACC
PDGFRa	F	CCT TGG TGG CAC CCC TTA C
	R	TCC GGT ACC CAC TCT TGA TCT T
SOX10	F	CCACGAGGTAATGTCCAACATG
	R	CAT TGG GCG GCA GGT ACT
GFAP	F	AGATCCGCACGCAGTATGA
	R	AGTCGTTGGCTTCGTGCTT
RPL37A	F	GTG GTT CCT GCA TGA AGA CAG TG
	R	TTC TGA TGG CGG ACT TTA CCG
